# Analysis of prices paid by low-income countries - how price sensitive is government demand for medicines?

**DOI:** 10.1186/1471-2458-14-767

**Published:** 2014-07-30

**Authors:** Divya Srivastava, Alistair McGuire

**Affiliations:** OECD, 2 Rue Andre Pascal, Paris, 75016 France; LSE Health, London School of Economics and Political Science, Houghton Street, London, WC2A 2AE United Kingdom

**Keywords:** Medicines, Low-income countries, Pharmaceutical policy, Health policy, Government procurement

## Abstract

**Background:**

Access to medicines is an important health policy issue. This paper considers demand structures in a selection of low-income countries from the perspective of public authorities as the evidence base is limited. Analysis of the demand for medicines in low-income countries is critical for effective pharmaceutical policy where regulation is less developed, health systems are cash constrained and medicines are not typically subsidised by a public health insurance system

**Methods:**

This study analyses the demand for medicines in low-income countries from the perspective of the prices paid by public authorities. The analysis draws on a unique dataset from World Health Organization (WHO) and Health Action International (HAI) using 2003 data on procurement prices of medicines across 16 low-income countries covering 48 branded drugs and 18 therapeutic categories. Variation in prices, the mark-ups over marginal costs and estimation of price elasticities allows assessment of whether these elasticities are correlated with a country’s national income.

**Results:**

Using the Ramsey pricing rule, the study’s findings suggest that substantial cross-country variation in prices and mark-ups exist, with price elasticities ranging from -1 to -2, which are weakly correlated with national income.

**Conclusions:**

Government demand for medicines thus appears to be price elastic, raising important policy implications aimed at improving access to medicines for patients in low-income countries.

## Background

Access to medicines is an important health policy issue. This paper considers demand structures in a selection of low-income countries from the perspective of public authorities. Analysis of the demand for medicines in low-income countries is critical for effective pharmaceutical policy where regulation is less developed, health systems are cash constrained and medicines are not typically subsidised by a public health insurance system.

The standard economic approach for measuring demand for a commodity is to calculate price elasticities, which requires data on prices and volumes. Low-income countries, in general, do not have robust data on prices and the quantities of medicines consumed. The estimation of price elasticities through conventional approaches is generally not possible and therefore, consequently there is little evidence on the price responsiveness either at the patient level or at the level of sales to government purchasers. There is a gap in basic empirical evidence that arises through an acute lack of data. Recent health related surveys have only begun to collect information on medicine prices but volume information is still lacking in many low-income countries.

For this study, information on procurement prices was accessible but volume information for sales to government purchasers was not. This data constraint limits the ability of policy makers to assess the impact that price regulation may have on the up-take of and access to medicines. To overcome the lack of volume information, information on prices and proxy estimates of price-cost mark-ups were used in conjunction with the general Ramsey formula to calculate the responsiveness of demand to product price. The use of Ramsey formula, allows demand responsiveness to be back-calculated as it is based on price-cost mark-ups, expressed as a fraction of price, being inversely related to their demand elasticities [1].

This model allows the pubic regulator to determine the optimal level of taxation of commodities to generate revenue, while trying to address distortions in the market. According to the Ramsey rule, a least distortionary tax is one where the tax is greatest on inelastic demands as this raises the consumer borne price over the marginal cost in inverse proportion to the elasticity of demand. The Ramsey pricing formula, commonly referred to as the inverse-elasticity rule, is:
1

where , the price elasticity of demand, , is the price-marginal cost mark-up over price, and *λ* is a constant (normally reflecting a total revenue constraint in general application). Before turning to the application of this rule, we give some background on the global pharmaceutical sector.

Due to the patenting of medicines, markets are characterised by the presence of a monopoly element. From the perspective of the firm, the pricing rules for a monopolist according to economic theory would see price set well above marginal cost.

Since prices of medicines in high-income countries would be unaffordable for low-income countries, the Ramsey pricing rule has been proposed as a potential policy response for low-income countries [2]. According to this rule, prices should be closer to marginal cost where the demand for medicines is more sensitive to price. Where demand is not sensitive to the medicine’s price, then price could be set at high levels to cross-subsidise low-income markets. From the firm’s perspective, if country markets are well segmented, intellectual property rights (IPRs) are protected globally and there is little threat of parallel trade or leakage into other country markets, Ramsey pricing could be used to establish affordability in different market environments. To implement Ramsey pricing however, the firm, requires adequate information about demand. Where such information is not forthcoming it has been proposed that a country’s income could be used as a proxy for a country’s price elasticity within the Ramsey approach to setting procurement prices in low-income countries [3].

The pharmaceutical industry is a global business and such cross-subsidisation strategies are an attractive mechanism to allow market exploitation across individual countries. Global sales in 2011 show that high-income regions such as North America (36%), Europe (24%) and Japan (12%) account for 72% of total pharmaceutical spending; branded drugs account for 63% of total pharmaceutical spending, but this is expected to decline as product development wanes and patents on existing products expire, leading to a rise in generic drug spending [4]. The pharmaceutical market is characterised as having high fixed costs, which may or may not be exclusively attributable to Research and Development (R&D). Some evidence suggests the average expenditure on R&D alone is $802 million per approved new drug [5]. R&D is considered an international activity (a fixed cost relative to the global market) because it can be located anywhere in the world and once the drug is developed, R&D expense is a sunk cost [6]. The remaining market access costs are specific to the country of sale and include distribution costs, marketing costs and interactions with government authorities for pricing and reimbursement negotiations [6].

These features of the pharmaceutical market highlight the important relationship between the pharmaceutical industry and the individual country regulators that purchase drugs on behalf of their population. High-income countries generate larger sales for the pharmaceutical industry with respect to volume and also have a higher degree of market power as a monopsonist when negotiating with firms due to the potentially high profit stream available in that country. Low-income countries are cash constrained, do not reflect high profit markets and as a result, do not have the same degree of buyer power in price negotiations.

The definition of pharmaceutical prices depends on where they occur in the supply chain (e.g. ex-manufacturer, or patient retail price). This study draws on upstream prices. Empirical work has commonly used upstream prices, such as ex-manufacturer prices, and country level measures of income [7–12]. One study found that besides per capita income, regulation played a critical role in explaining global price variation [7]. Furthermore, direct price control measures were found to result in an average 20% price reduction. Policies such as procurement through a central government agency, and promotion of generics also contributed to lowering the general price level of pharmaceuticals.

Other work has found that per capita income helped to explain global price differences, but that this relationship weakened over time as pharmaceutical firms offered discounts within individual countries that were unrelated to per capita income [8]. A relationship between price and income was found in high-income countries only [9, 11]. In less affluent countries in Latin America for example, high drug prices appear to partly reflect the skewed income distribution of income and the manufacturer’s tendency to target prices to the affluent minority [11]. Recent work on a select number of middle and low-income countries analysed the determinants of ex-manufacturer prices for originator and generic drugs as well as retail prices for drugs to treat HIV/AIDS, TB and malaria [12]. The study concludes that an income elasticity which ranges from 0 to 0.10 suggests that drugs are unaffordable because within-country income contributes to relatively high prices. Tendered procurement using data from large non-government organisations imposes quality standards and reduces the prices of originator and generic drugs compared with their respective retail pharmacy prices. Overall drug prices remain unaffordable for the majority population, contributing to a lower per capita use of drugs in these countries.

Descriptive analysis has also found differences in prices for the same drug across countries with similar income levels [13–15] and within countries [16]. The vast majority of the literature has thus concentrated on the relationship observed across readily available data on price and income.

The aim of this paper is to provide complementary evidence on the pattern of demand in low-income countries through the estimation of price elasticities. The focus of the study is on government behaviour and buying power. The interest is to study and understand government behaviour regarding drug procurement, with price being the main response variable. The study uses government procurement (upstream) price data at the molecule level or price per pill that was aggregated up to pack size, drawing on a large cross-sectional sample of low-income countries. The study has three objectives: to explore the variation in pharmaceutical prices and price mark-ups; to investigate the price sensitivity at the government level and compute price elasticities for sales to government purchasers; and to analyse the relationship between these estimated price elasticities and income.

## Methods

The empirical approach to calculate price elasticities adopted the formulation of Ramsey pricing given as:
2

where  is the inverse elasticity of demand, the procurement pack price of the branded medicine is Pj for medicine j, and MCj is the marginal production cost for product j. A true estimate of the marginal cost (MCj) of producing a given drug is not available so the pack price of the available generic substitute medicine was used as a proxy estimate of the marginal cost. Theory assumes that once generic firms enter the market, the price of the medicine falls and approaches to marginal cost, as the number of generic firms increases [17].

The model assumes that pharmaceutical firms are profit maximisers, they have fixed costs, break-even (hence the inverse elasticity formula has no constant in the numerator on the right hand side of (2), and our back-calculations are lower end estimates), and marginal costs are not zero. The model further assumes that cross-price elasticities for branded products are zero, and that there are no perfect complements. It is also assumed that for branded products there remains a monopoly element, that price is related to demand and that firms are aware of product price-cost mark-ups.

The left hand side of equation (2) estimates the differences between price and marginal cost as a fraction of price. The left hand side of the equation should be inversely related to the demand elasticity. Prices were kept at the presentation level to provide price elasticity estimates at the molecule level.

### Data and variables

**Figure 1:**
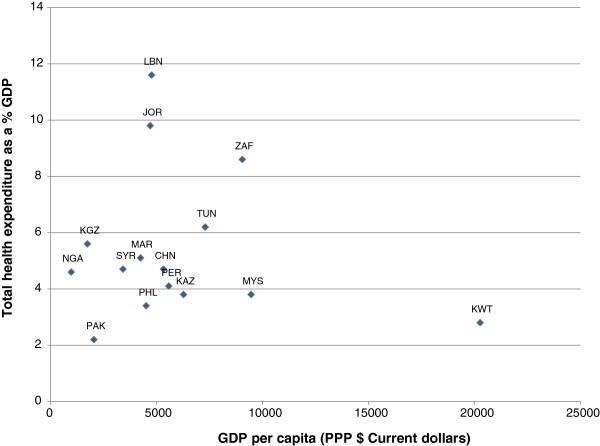
**Total health expenditure % GDP and GDP per capita, 2003.**

The dataset comes from the World Health Organization (WHO) and Health Action International (HAI) database for one year, 2003 [18]. The price information covers 18 therapeutic areas and 48 branded drugs in 16 countries: China (sampled in Shandong and Shanghai), Jordan, Kazakhstan, Kuwait, Kyrgyzstan, Lebanon, Malaysia, Morocco, Nigeria, Pakistan, Peru, Philippines, South Africa (region of Kwa-Zulu-Natal), Syria, Tunisia, and Uganda. Government procurement price for the originator branded drug in each country is used. All prices were provided in US dollars for the year 2003.By way of background the countries in the sample had GDP per capita that ranged from 982 $ current international dollars in Nigeria to 20280 in Kuwait as shown in Figure 1. Countries also varied in how much they spent on health as a share of GDP from 2.2% in Pakistan to 11.6% in Lebanon.

Procurement prices are the prices that governments and other central purchasers pay to procure medicines, and are generally obtained through a tendering process. The procurement prices for the public sector are either collected in the administrative centre (procurement offices or central medical stores). In a few situations, the procurement prices included local taxes and handling charges [18]. The data on procurement come from central or regional authorities or the Ministry of Health for 9 out of the 16 countries. Four out of the 16 countries used a combination of data from both procurement authorities and government affiliated public hospitals, while the remaining four collected procurement data from either government hospitals, or tenders from wholesalers.

Prices for each country are presented as the median price at the presentation level: drug molecule name; pack size and strength. The 18 therapeutic areas and 48 drugs covered are: antacids (2); antibiotics (6); antifungal (3); antihistamine (1); anti-infective (1); anti-inflammatory (2); anti-parasitic (2); antiviral (4); asthma (2); cardiovascular disease (14); contraceptive (1); diabetes (3); and nervous system disorders (7). The top therapeutic categories with the most number of observations, (17 for antihypertensives and antibiotics; 15 for epileptic drugs), are found in Table 1.

**Table 1:** **Summary of drug data**

Molecule name	Therapeutic category	Observations	Dose	Countries
Carbamazepine	Epilepsy	8	200 mg	China, Kazakhstan, Kuwait, Malaysia, Morocco, Philippines, Syria
Ceftriaxone	Antibiotic	7	1 g	China, Kazakhstan, Malaysia, Morocco, Philippines, South Africa
Salbutamol	Asthma	7	0.1 mg	China, Kazakhstan, Kuwait, Morocco, Tunisia, Uganda
Fluoxetine	Antidepressant	6	20 mg	China, Jordan, Malaysia, Philippines, Tunisia
Metformin	Diabetes	6	500 mg	China, Morocco, Nigeria, Pakistan, Philippines
Aciclovir	Antiviral	5	200 mg	Jordan, Kazakhstan, Philippines, Syria, Tunisia
Amitriptyline	Antidepressant	5	25 mg	Jordan, Lebanon, Morocco, Syria, Tunisia
Captopril	Antihypertensive	5	25 mg	Kazakhstan, Malaysia, Morocco, Pakistan, Philippines
Ciprofloxacin	Antibiotic	5	500 mg	Kazakhstan, Morocco, Nigeria, Philippines, South Africa
Diclofenac	Anti-inflammatory	5	25 mg	China, Kazakhstan, Morocco, Philippines, Syria
Phenytoin	Epilepsy	5	100 mg	Jordan, Kuwait, Lebanon, Malaysia, Tunisia
Beclometasone	Asthma	4	50 mcg	China, Morocco, Peru
Diazepam	Anxiolytic	4	5 mg	Jordan, Morocco, Syria, Tunisia
Losartan	Antihypertensive	4	50 mg	China, Kazakhstan, Malaysia
Omeprazole	Antacid	4	20 mg	China, Philippines, South Africa
Ranitidine	Antacid	4	150 mg	Kazakhstan, Nigeria, Philippines,Syria
Fluconazole	Antifungal	3	200 mg	South Africa, Tunisia, Uganda
Fluphenazine	Antipsychotic	3	25 mg	Jordan, Morocco, Peru
Indinavir	Antiviral	3	400 mg	Lebanon, Malaysia, Morocco
Loratadine	Antihistamine	3	10 mg	China, Malaysia, Syria
Simvastatin	Lipid lowering	3	20 mg	China, Jordan, Malaysia
Zidovudine	Antiviral	3	100 mg	Lebanon, Malaysia, Morocco
Amlodipine	Calcium channel blocker	2	5 mg	China, Malaysia
Atenolol	Antihypertensive	2	50 mg	Philippines, Syria
Co-trimoxazole	Antibiotic	2	8 + 40 mg/ml	Syria, Tunisia
Fluconazole	Antifungal	2	150 mg	Jordan, Kazakhstan
Furosemide	Diueretic	2	40 mg	Jordan, Philippines
Mebendazole	Antiparasitic	2	100 mg	Kazakhstan, Kyrgyzstan
Metronidazole	Antiparasitic	2	500 mg	Philippines, Syria
Nevirapine	Antiviral	2	200 mg	Lebanon, Morocco
Nifedipine retard	Anti hypertensive	2	20 mg	Kuwait, Morocco
Pyrazinamide	Antiinfectives	2	500 mg	Morocco, Philippines
Valproic acid	Epilepsy	2	200 mg	Malaysia, Morocco
Acetylsalicylic acid	Anti-inflammatory	1	NA	Morocco
Amoxicillin	Antibiotic	1	250 mg	Jordan
Benzathine benzylpenicillin	Antibiotic	1	1.2 MIU vial	Morocco
Cefradine	Antibiotic	1	NA	China
Chloroquine	Antimalarial	1	NA	Tunisia
Cimetidine	Antacid	1	NA	China
Digoxin	Cardio therapy	1	0.25 mg	Philippines
Diltiazem	Calcium channel blocker	1	60 mg	Jordan
Enalapril	Antihypertensive	1	20 mg	Jordan
Glibenclamide	Diabetes	1	5 mg	Philippines
Gliclazide	Diabetes	1	NA	China
Insulin neutral	Diabetes	1	100 ml	Kuwait
Isosorbide dinitrate	Cardio therapy	1	10 mg	Philippines
Itraconazole	Antifungal	1	100 mg	Malaysia
Lisinopril	Antihypertensive	1	10 mg	Kuwait
Medroxyprogesterone	Contraceptive	1	150 mg	Kazakhstan
Methyldopa	Antihypertensive	1	250 mg	Jordan
Paracetamol	Anti-inflammatory	1	500 mg	Syria
Prazosin	Antihypertensive	1	1 mg	Malaysia
Streptomycin	Antibiotic	1	1 g vial	Morocco

Source: WHO/HAI 2006.

Note Due to lack of data, price elasticities could not be calculated for the following: Acetylsalicylic acid; Cefradine; Chloroquine; Cimetidine; and Glicazide”.

Note: Data from China were sampled in two regions, which resulted in two observations for this country. The corresponding elasticities were calculated separately.

Data on marginal costs were required, but these were unavailable for branded drugs. The closest proxy available was the price of the relevant generic drug in the national market. This information implies that for the majority of countries, the branded drugs studied were off-patent. For a small number of drugs, generic product data were used, and for the remaining, the average international procurement price was used as a proxy. Information on these average prices was supplied from Management Science for Health (MSH). MSH maintains a database of international procurement prices offered by international suppliers to low-income countries. This dataset is a standard source of international procurement prices and is considered a gold standard [19].

In this study, two-thirds of the observations used the MSH reference price where there was no generic in the market and one-third of the observations used a generic price that was available in the market to calculate the price elasticity. There were, however, no systematic differences (e.g. by therapeutic category or by country) in the computed price elasticities between the set of data that use the MSH price compared with the generic price. The cross-sectional nature of this study cannot capture the lagged or residual price of effect when there are generic equivalents in the market. One reason might be that generics in these country settings are not real substitutes and do not act as a constraint as observed in high-income countries. In middle and low-income these settings, there is evidence to suggest that generics are perceived as low quality [12]. This suggests that other factors could drive price differences between originators and their equivalents, such as regulatory skill and procurement ability.

While the WHO/HAI survey attempted to collect price information on the same drug in each country, this was not always possible. A final total of 139 observations were therefore available for analysis. In the data sample, the highest number of countries with the same drug was 7 for carbamazepine (treatment of epilepsy), and 6 for both ceftriaxone (antibiotic) and salbutamol (treatment of asthma).

## Results and discussion

### Descriptive statistics

**Figure 2:**
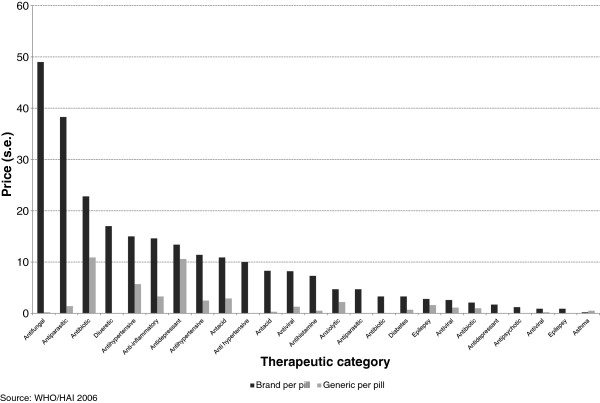
**Standard error in prices per pill.**

Prices of medicines show significant variation by therapeutic class of drugs and even within therapeutic classes across countries. Medicines are typically available in pack (e.g. pack of 25 tablets, 50, etc.). Branded prices sold according to their packs ranged from US$ 325 (fluconazole in Tunisia, and zidovudine and nevirapine in Lebanon) to less than a US$ 1. Antiviral drugs had the highest prices per pack while most antibiotics (except for ciproflaxin) were the least expensive for both branded and generics. Most medicines were priced at less than US$ 50 with Jordan and Kazakhstan having the lowest prices. The top prices of generics per pack ranged from US$ 162 (indinavir, zidovudine, nevirapine in Morocco, Malaysia and Lebanon, respectively) to less than US$ 1. Most medicines were priced less than US$ 10, with Kazakhstan and Kyrgyzstan having the lowest prices.Price comparisons of medicines are difficult when pack sizes vary. Prices can be normalised by taking the unit price which allows for more straightforward comparisons. The wide variability in prices per pill was examined by calculating the standard error for each molecule. Figure 2, which shows the standard error in prices by pill, reveals wide variations for certain antifungal and antibiotics drugs. A similar pattern was observed by pack size (not shown).

**Figure 3:**
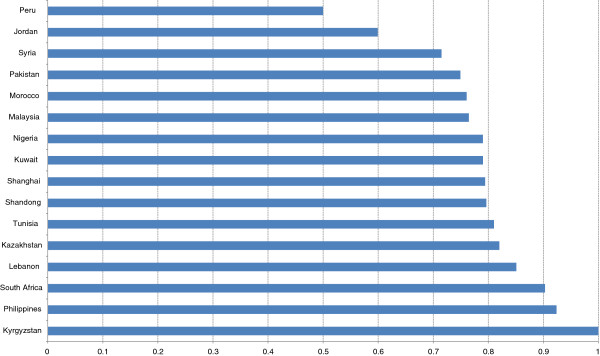
**Average mark-ups across countries.**

Mark-ups were computed for each drug and averaged for each country. The data indicate that mark-ups vary across the countries ranging from 50% to 100% with Peru (50%), Jordan (60%) having the lowest and South Africa, Philippines and Kyrgyzstan having the highest (90% to 100%) as shown in the Figure 3. Most countries have average mark-ups ranging between 70% and 80%. Unregulated mark-ups along the supply chain have shown to contribute to high retail prices of medicines in low-income countries [18, 20].

### Price elasticity estimates

**Figure 4:**
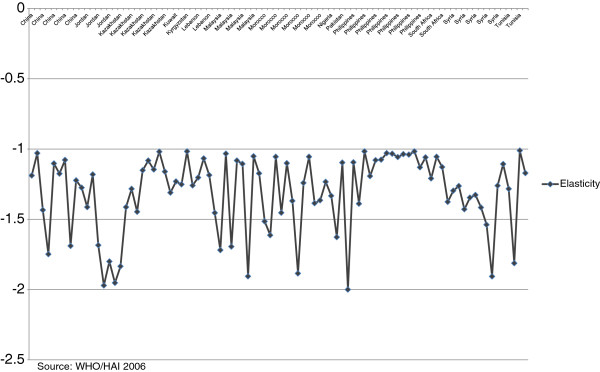
**Price elasticity by country.**

Price elasticities were back-calculated using the Ramsey rule as shown in equation (2). Details are found in Table 2. Estimates of the price elasticities for different therapeutic products and countries range from between -1 to -2. These findings suggest that if the procurement price of the drug increases by 10%, demand for the drug would drop by 10% to 20%. This implies that low-income countries are more responsive to changes in the prices of medicines and, assuming these estimates are a good first approximation, certainly more responsive than high-income countries.Figure 4 displays the across country estimates. Similar results show that across drugs, estimates are also fairly consistent. The outliers appear random, which suggests no systematic bias in the results, either by country or by drug.

**Table 2:** **Elasticity results by molecule name**

Molecule name	Therapeutic category	Country	Elasticity	Brand pack price ($US)	Generic pack price ($US)	Pack size
Aciclovir	Antiviral	Kazakhstan	-1.3	17.5	3.9	25
Aciclovir	Antiviral	Tunisia	-1.1	25.0	2.4	25
Aciclovir	Antiviral	Philippines	-1.1	32.8	2.4	25
Aciclovir	Antiviral	Syria	-1.3	21.8	5.0	25
Amitriptyline	Antidepressant	Jordan	-1.4	2.6	0.8	100
Amitriptyline	Antidepressant	Morocco	-1.2	5.1	0.8	100
Amitriptyline	Antidepressant	Lebanon	-1.3	3.4	0.7	100
Amlodipine	Calcium channel blocker	Malaysia	-1.1	8.8	0.4	30
Atenolol	Antihypertensive	Syria	-1.4	5.9	1.7	60
Atenolol	Antihypertensive	Philippines	-1.0	7.4	0.3	28
Beclometasone	Asthma	Peru	-2.0	6.8	3.4	200
Beclometasone	Asthma	Morocco	-1.5	7.9	2.5	200
Benzathine benzylpenicillin	Antibiotic	Morocco	-1.4	2.2	0.6	4
Captopril	Antihypertensive	Morocco	-1.5	59.6	20.3	60
Captopril	Antihypertensive	Malaysia	-1.7	3.9	1.6	60
Captopril	Antihypertensive	Kazakhstan	-1.4	5.1	1.6	60
Captopril	Antihypertensive	Pakistan	-1.1	5.6	0.5	60
Captopril	Antihypertensive	Philippines	-1.1	56.2	4.0	150
Carbamazepine	Epilepsy	Kazakhstan	-1.8	26.9	12.2	150
Carbamazepine	Epilepsy	Shanghai	-1.2	13.1	2.0	100
Carbamazepine	Epilepsy	Shandong	-1.2	12.5	2.0	100
Carbamazepine	Epilepsy	Philippines	-1.1	115.1	10.0	500
Carbamazepine	Epilepsy	Kuwait	-1.3	12.2	2.9	150
Carbamazepine	Epilepsy	Malaysia	-1.5	6.4	2.0	100
Carbamazepine	Epilepsy	Syria	-1.4	20.2	5.5	150
Ceftriaxone	Antibiotic	South Africa	-1.2	8.5	1.5	1
Ceftriaxone	Antibiotic	Malaysia	-1.7	6.1	2.6	1
Ceftriaxone	Antibiotic	Kazakhstan	-1.4	10.4	3.0	1
Ceftriaxone	Antibiotic	Philippines	-1.4	9.1	2.6	1
Ceftriaxone	Antibiotic	Shanghai	-1.1	10.2	0.7	1
Ceftriaxone	Antibiotic	Shandong	-1.0	12.5	0.4	1
Ciprofloxacin	Antibiotic	Kazakhstan	-1.2	0.2	0.0	1
Ciprofloxacin	Antibiotic	Nigeria	-1.3	0.9	0.2	1
Ciprofloxacin	Antibiotic	Morocco	-1.6	2.1	0.8	1
Ciprofloxacin	Antibiotic	Philippines	-1.0	111.0	3.2	100
Ciprofloxacin	Antibiotic	South Africa	-1.1	0.7	0.0	1
Co-trimoxazole	Antibiotic	Syria	-1.5	0.8	0.3	70
Co-trimoxazole	Antibiotic	Tunisia	-1.2	1.9	0.3	70
Diazepam	Anxiolytic	Tunisia	-1.8	2.8	1.3	100
Diazepam	Anxiolytic	Jordan	-1.7	0.9	0.4	100
Diazepam	Anxiolytic	Syria	-1.4	3.9	1.2	100
Diazepam	Anxiolytic	Morocco	-1.1	3.8	0.4	100
Diclofenac	Anti-inflammatory	Shandong	-1.7	9.3	4.0	100
Diclofenac	Anti-inflammatory	Syria	-1.3	9.3	1.9	100
Diclofenac	Anti-inflammatory	Philippines	-1.0	15.6	0.5	100
Diclofenac	Anti-inflammatory	Kazakhstan	-1.1	27.1	2.1	100
Diclofenac	Anti-inflammatory	Morocco	-1.1	9.7	0.5	100
Digoxin	Cardio therapy	Philippines	-1.1	28.5	3.3	500
Fluconazole	Antifungal	South Africa	-1.1	107.7	12.3	30
Fluconazole	Antifungal	Tunisia	-1.0	325.9	3.6	30
Fluconazole	Antifungal	Jordan	-1.8	0.2	0.1	1
Fluoxetine	Antidepressant	Malaysia	-1.0	27.5	0.9	30
Fluoxetine	Antidepressant	Shandong	-1.4	34.6	10.5	30
Fluoxetine	Antidepressant	Shanghai	-1.7	35.1	14.3	30
Fluoxetine	Antidepressant	Philippines	-1.0	49.7	0.8	28
Fluphenazine	Antipsychotic	Morocco	-1.4	1.8	0.5	1
Fluphenazine	Antipsychotic	Jordan	-2.0	1.0	0.5	1
Furosemide	Diueretic	Philippines	-1.0	3.5	0.1	28
Furosemide	Diueretic	Jordan	-2.0	0.2	0.1	20
Glibenclamide	Diabetes	Philippines	-1.1	14.6	0.8	200
Indinavir	Antiviral	Morocco	-1.9	133.4	62.6	180
Loratadine	Antihistamine	Syria	-1.3	4.2	1.0	20
Loratadine	Antihistamine	Malaysia	-1.1	2.4	0.2	10
Mebendazole	Antiparasitic	Kazakhstan	-1.0	1.3	0.0	6
Mebendazole	Antiparasitic	Kyrgyzstan	-1.0	1.5	0.0	6
Medroxyprogesterone	Contraceptive	Kazakhstan	-1.2	7.4	1.0	1
Metformin	Diabetes	Nigeria	-1.2	7.1	1.4	100
Metformin	Diabetes	Pakistan	-1.6	1.7	0.7	100
Metformin	Diabetes	Shanghai	-1.2	15.3	2.8	100
Metformin	Diabetes	Philippines	-1.2	11.0	1.8	100
Metronidazole	Antiparasitic	Syria	-1.9	0.8	0.4	20
Metronidazole	Antiparasitic	Philippines	-1.0	24.5	0.4	100
Nevirapine	Antiviral	Lebanon	-1.2	197.8	31.0	60
Nevirapine	Antiviral	Morocco	-1.2	72.3	14.1	60
Nifedipine Retard	Anti hypertensive	Morocco	-1.1	41.2	2.2	100
Nifedipine Retard	Anti hypertensive	Kuwait	-1.3	11.9	2.4	100
Omeprazole	Antacid	Shandong	-1.1	39.1	3.6	30
Omeprazole	Antacid	Shanghai	-1.3	39.2	8.5	30
Paracetamol	Anti-inflammatory	Syria	-1.3	1.1	0.2	20
Phenytoin	Epilepsy	Lebanon	-1.2	4.1	0.7	100
Phenytoin	Epilepsy	Kuwait	-1.2	3.7	0.7	100
Phenytoin	Epilepsy	Jordan	-1.2	4.6	0.7	100
Phenytoin	Epilepsy	Tunisia	-1.3	3.2	0.7	100
Ranitidine	Antacid	Philippines	-1.1	23.0	1.2	50
Ranitidine	Antacid	Kazakhstan	-1.1	11.7	1.5	60
Ranitidine	Antacid	Syria	-1.3	9.5	2.4	60
Simvastatin	Lipid lowering	Malaysia	-1.1	104.2	10.0	120
Streptomycin	Antibiotic	Morocco	-1.4	0.4	0.1	1
Zidovudine	Antiviral	Lebanon	-1.1	296.0	18.6	150
Zidovudine	Antiviral	Malaysia	-1.9	78.8	37.5	100

Source: WHO/HAI 2006.

Out of 139 observations, 49 further estimates were dropped for two reasons. In the first case, observations where the branded price was below the generic price back-calculation of elasticity was not possible (19 observations). Second, a number of implausible estimates of price elasticities ranging between -3 and -27 (30 observations) arose, where the branded and generic pack price were relatively similar in value; probably indicating that at least in these cases the generic price was not a reasonable approximation to marginal cost. While this is not an insignificant reduction in the sample size, the pattern and range of elasticities remained consistent across drugs and across countries.

All of the dropped observations used the MSH reference price where there was no generic in the national market. There was no clear pattern among the therapeutic categories as almost all therapeutic categories were affected. Only five therapeutic categories did not have observations that were dropped: anxiolytics, diuretics, antiparasitic, contraceptive and anti-inflammatory medication. Only a few countries were disproportionately affected. Uganda had 2 observations in the data set and both were outliers. Jordan and Peru, had similar numbers of computed elasticities that were both in the normal range and considered outliers. Seven countries: China (Shandong province), Kazakhstan, Kuwait, Malaysia, Morocco, Nigeria, and Tunisia had some outliers but the majority their observations had computed elasticities in the reported normal range.

Sensitivity analysis was carried out on the results. First, generic prices, which were used as proxies for marginal cost, were varied to see if the results would significantly change the results: they were increased and decreased ranging from 5% to 33%. The results showed that estimates stayed within the original range with very few changes in the country and drug specific results.

### Income correlation

Correlations were calculated between price and measures of income: Gross domestic product (GDP) per capita, and gross national income (GNI) per capita [21]. Correlations between price and health care expenditure were also assessed and are shown in Table 3. Three expenditure measures were used: per capita public health expenditure (PHE); total health expenditure (THE) as a % of GDP; and per capital total health expenditure.

**Table 3:** **Correlations between price and income, price and expenditure**

	GDP per capita	GNI per capita	PHE per capita	THE % GDP	THE per capita
Pack price	-0.000 (0.59)	-0.011 (0.55)	0.066 (0.72)	0.120 (0.10)	0.215 (0.05)
Price per pill	0.007 (0.83)	-0.004 (0.97)	0.008 (0.35)	-0.022 (0.62)	0.050 (0.18)

Note: P-value in parentheses. Source: WHO/HAI 2006; World Bank Development Indicators 2005.

**Figure 5:**
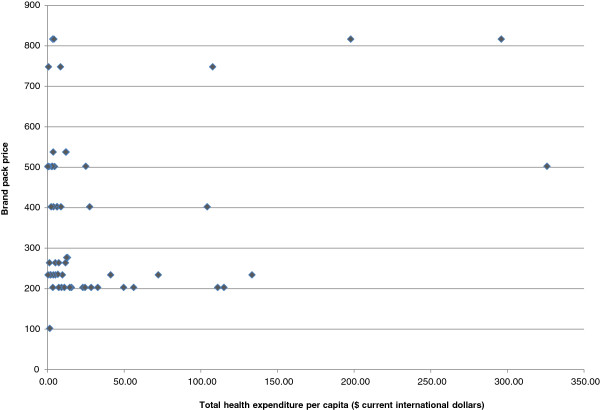
**Relationship between brand pack price and total health expenditure per capita, 2003.**

The results suggest little relationship with income measures: -0.01 to 0.007 (GDP); -0.011 to -0.004 (GNI) and a weak relationship with expenditure measures (0.008 to 0.2) and some significance with total health expenditure per capita. This result is not consistent with some findings where a positive association between a country’s income and price was found [7, 8], but is consistent with a recent study, which might suggest a change in recent global pricing practices [22]. A small positive relationship between government health expenditure and the price of the drug was also found as shown in Figure 5. This implies that higher government expenditure on health is related to having higher priced drugs. These results have intuitive appeal and are consistent with the general findings in the literature [23] Figure 5.

### Limitations

While the study was initiated to attempt to estimate a wide range of pharmaceutical product price elasticities in low-income countries where data, especially on volumes, is often limited the limitations should be noted. The analytical approach assumes that firms pay some attention, at least implicitly, to the Ramsey pricing rule, but this may not be the case. Second, the results assume that the proxy used for marginal cost, generic prices or international procurement prices are reliable measures. In fact a number of observations had to be excluded from the analysis where the use of the proxy returned inadmissible estimates. Further, not all prices gained from the survey reflect true government procurement prices. As noted, a small minority of cases drew on data from government operated hospitals. Furthermore, elasticities were calculated using standardised pack sizes which may not necessarily be representative of pack sizes in each country.

Nevertheless, this study is an exploratory exercise and the adopted analytical approach ought to be viewed against the substantial data constraints faced in estimating demand curves for pharmaceutical products in low-income countries. Even indirect methods of estimation prove useful in returning empirical estimates of demand responsiveness were severe data constraints exist.

## Conclusion

The aim of this paper was to understand the pattern of pharmaceutical prices across countries and country price responsiveness. The findings indicate that price elasticities at the government level range between –1 and –2 across all therapeutic classes studied. Sensitivity tests found that the results stayed within this range. While the technique required a number of assumptions to undertake a back-calculation to overcome data restrictions on product volumes, these estimates are a first attempt at better understanding demand structures in these settings and should be viewed as suggestive.

That said the evidence presented here suggests that the price response of low-income countries to pharmaceutical price, when the product is centrally procured, is robustly elastic. Moreover there seems little relationship with a countries income, although some correlation with health care expenditure levels. Taken together this evidence would suggest that if pharmaceutical manufacturers do not price discriminate on the basis of ability to pay, low-income countries will face market access restrictions to new products where the global pharmaceutical policy is aimed to recover high R&D costs.

Possibly as a response to market access restrictions, explicit pricing policies are not common place in low-income countries. Such policies are involved and incur administration costs [23]. A WHO report noted that such costs contribute to the low uptake of adopting pricing policies with only half of all low-income countries having any pricing policy in place [24, 25]. Of course the implementation of pricing policies requires the use of reliable data and it is imperative that procurement agencies begin the task of collecting reliable data on both price and volume in the pharmaceutical sector—international bodies already involved in procurement for low-income countries could play a key role. Without such data, policy objectives cannot be implemented or assessed.

As noted by this study, information on volume would provide better estimates of price elasticities. A properly devised longitudinal study would allow for the analysis of patterns in the demand for medicines over time. Data collection relating to regulatory and supply issues would provide insight in the policy implications of pricing and reimbursement and licensing decisions. In some settings government procurement could play a small role in medicine access relative to non-governmental bodies and would shed light on the interaction between these actors [19, 26–28]. Recent efforts by the WHO/HAI, Access to Medicines index, Medicines Transparency Alliance (MeTA) signal an important priority shift in this area. This study has shown that access to medicines is a pressing yet complex public health issue. Research in this area is needed to continue to build evidence to inform the design of effective pharmaceutical policy and to contribute to improving access to medicines for people in low-income countries.

### Ethics

Ethics approval statement not required for analysing the publicly available data on prices of medicines. Health Action International and the World Health Organization make this data freely available and accessible to researchers.

## Abbreviations

HAI: Health action international
R&D: Research and development
WHO: World health organization.
